# Practical Notes and Formulæ

**Published:** 1880-09-20

**Authors:** 


### PRACTICAL NOTES AND FORMULÆ.

**Wine of Tar—Excellent for Chronic Coughs.**—As usually made, wine of tar is an unsightly, unstable and unpalatable article, but as prepared by the following receipt will be found free from these objections :

| | | |
|---|---|---|
| ℞ | Tar | 4 troyounces |
| | Granulated sugar | 5 troyounces |
| | California sherry | 1½ pints |
| | Water, sufficient quantity to make | 2 pints |
| | Sand, washed and dried | 8 ounces |

Rub the tar with the sugar and sand in a mortar, then with the wine and water. Pour into a bottle all the ingredients and agitate occasionaily for 4 or 5 days. Filter with paper pulp, when a fine clear wine will result, highly impregnated with the tar, which will keep without undergoing acetous fermentation. By employing the California wine and water a preparation results that does not contain much more alcohol than would be the case if made by the ordinary plan. The employment of sand is on account of the mechanical division it effects. —*Amer. Journal of Pharmacy.*

**To Disguise Castor Oil.**—

| | | |
|---|---|---|
| ℞ | Syrup of orange peel | f℥ j |
| | Syrup of gum arabic | f℥ ss |
| | Caramel | fʒ j |
| | Tartaric acid | xxv grains |
| | Water | f℥ iv |

Mix.

**Saint Barthelemy's Fever Liniment.**—Dr. Sezeric makes this liniment by the following process :

| | | |
|---|---|---|
| ℞ | Olei terebinthinæ | 125.0 |
| | Tincture opii | 5.0 |
| | Camphoræ | 3.0 |
| | Olei olivæ | 60.0 |

M. S. Apply for 6 minutes every 6 hours to the whole spine. After applying three to four times the intermittent fever stays away.—*Pharm. Centralb.*

**Benzoate of Soda.**—Dr. Cady, of Wisconsin, in N. Y. Record, recommends the following as very useful in diphtheria and pneumonia

| | | |
|---|---|---|
| ℞ | Sodii benzoat | ʒ ij |
| | Aqua mentha pip | ℥ ss |
| | Syrup aurantii | ℥ ss |
| | Aqua dest | ℥ iij |

M. S. Dose, a tablespoonful every three hours.

**Diphtheria.**—It is somewhat amusing to watch the changes and differences in medical opinion in regard to the treatment of this much dreaded disease. Dr. N. F. Brown, in the Detroit Lancet, writes an article recommending very highly the following treatment, which has a rather familiar look, and, if we are not mistaken, was in use twenty years ago. Its age is not against it, however. The novelty of practice often brings disappointment, and we have to fall back upon the old treatment :

When called to see a child, three or four years ago, I gave the following prescription :

| | | |
|---|---|---|
| ℞ | Chlorate of potash | ʒ ss |
| | Pure glycerine | ʒ iij |
| | Mur. Tinct. iron | gtt- xx |
| | Water, add., q. s | ʒ iij |

M. Sig. Give a teaspoonful every half hour, day and night, arousing the patient when necessary to give the medicine punctually, and when the medicine is all used, renew the prescription, and give the same dose every hour, instead of half-hour. Follow this with tonics, iron quinia, strychnia, etc.—*Boston Med. Journal.*

**Hæmoptysis.**—Dr. Pepper, in a lecture at Philadelphia Hospital, says :

Of drugs, ergot seems to be the most powerful in checking hæmoptysis. The extractum ergotæ fluid. may be given in doses of a teaspoonful every fifteen minutes, until the hemorrhage is stopped, and then continued in smaller doses, or it may be given by hypodermic injection, in doses of gtt. xv, or ergotine may be used. If the stomach is irritable, gr. v of ergotine may be given per rectum. Sometimes ergot will have no appreciable effect. Under such circumstances I think that gallic acid is the next best remedy. I frequently combine it with aromatic sulphuric acid, which makes a more efficient and pleasant mixture.

| | | |
|---|---|---|
| ℞ | Acidi gallici | ʒ ij |
| | Acidi sulphurici aromat | f. ʒ j |
| | Glycerinæ | f. ℥ j |
| | Aquæ, q. s. ut ft | f. ℥ vj M |

Dose, a tablespoonful, as required.

This is to be given every hour, every half-hour, or at shorter intervals until the hemorrhage is brought under control. This, I think, ranks next to ergot, and where the stomach refuses ergot, or where ergot produces no effect, I usually resort to this combination.

**Hoof Ointment.**—Very satisfactory results are said to have been obtained with the following ointment in the treatment of all diseases peculiar to hoofs, such as cracks, scratches, cuts, etc. :

℞ Benzoin catechu, bone charcoal, each ............... 10 parts

Reduce to a fine powder and add :

| | |
|---|---|
| Pure carbolic acid | 8 parts |
| Petroleum ointment | 100 parts |
| Yellow wax | 10 parts |

Mix, with a gentle heat.—*Pharm. Centralb.*

### Artificial Selter's Water.—

℞ Chloride calcium.................................. gr. iv

Chloride magnesium.................................. gr. xij

Chloride sodium.................................. gr. xv

Citrate iron.................................. gr. ss

Tartaric acid.................................. ʒ ij

Bicarbonate soda.................................. ʒ ijss

Water.................................. q. s.

Dissolve all the salts but bicarb. soda and the tartaric acid in a pint of water; pour into a champagne bottle, and fill up with water until the bottle lacks two ounces of being full; add the soda and acid, and cork at once; wire the cork, and allow the bottle to stand for six hours.—*Med. Herald.*

**A Palatable Solution of Salicylic Acid.**—Dr. Flexner, of Louisville, recommends the following combination:

℞ Salicylic acid.................................. ℥j—℈ viij

Citrate of potash.................................. ℥ ij

Glycerine.................................. ℥ viij

Simple elixir q. s. ad.................................. Oj

The citrate of potash is to be dissolved in the glycerine by the aid of a gentle heat, after which the acid is to be stirred in, and a gentle heat maintained until it is completely dissolved. On cooling, simple elixir is to be added to bring it up to the required measurement, after which the solution is to be strained. It contains five grains of salicylic acid to the drachm, and is miscible in all proportions with water without the separation of any acid.—*Mich. Med. News.*

**Arsenic in Neuralgia.**—Dr. J. T. Stewart states, in the Peoria Medical Monthly, that he has been in the habit, for many years, of prescribing arsenic combined with opium in ordinary neuralgia, and found it of great value. His formula is as follows:

℞ Liquor potas. arsen.................................. ʒ iij

Tinct. opii.................................. ʒ iss

Alcohol.................................. ʒ iss M

Sig. Give ten drops in water every three hours, increasing the dose by two or three drops daily until the disease is controlled, or until decided constitutional effects are produced.—*Med. and Surg. Rep.*

### Aristocratic Remedy for Itch.—

℞ Balsam of Peru.................................. 1 ounce

Benzoic acid.................................. 110 grains

Oil of cloves.................................. 40 drops

Alcohol.................................. 2½ drachms

Simple cerate.................................. 7 ounces.

Dissolve the essential oil and the benzoic acid in the alcohol, and mix them with the cerate. Lastly, add the balsam of Peru. It is said to effect a cure in twenty-four hours.—*Med. and Surg. Rep.*

**Dyspepsia and Consumption.**—Glycerine in combination with iodine is recommended as a substitute for cod-liver oil in strumous cases. The following is suggested as an admirable tonic in cases of lung affection attended, as is often the case, with indigestion :

| | | |
|---|---|---|
| ℞ | Pure glycerine | ℥ ij |
| | Tinct. iodine | ʒ ss |
| | Iodii potass | gr. vi |
| | Tinct. nux vomica | ʒ j |
| | Syr. wild cherry | ℥ ij |

Dose, a dessert spoonful before each meal and at bed time if the cough be troublesome.

**For the Removal of Tan and Freckles.**—Dr. J. Nevins indorses the formula of Prof. White.

| | | |
|---|---|---|
| ℞ | Hydrarg. bichlorid | gr. vj |
| | Acid. muriat. dil | ʒ j |
| | Aqua | ℥ iv |
| | Alcohol | ℥ ij |
| | Aq. rosæ | ℥ ij |
| | Glycerine | ℥ j |

Mix. Apply at night, and wash off the skin with soap in the morning.—*Chicago Medical Review.*

**Simple Elixir.**—It may be prepared by mixing :

| | | |
|---|---|---|
| ℞ | Spirit of orange | ʒ ij |
| | Spirit of cinnamon | gttx |
| | Alcohol | ℥ iv |
| | Simple syrup | ℥ vj |
| | Water | ℥ vj |

**For Barber's Itch.**—Brame recommends the following treatment: Shave off the hairs or cut them very short; then apply, once or twice a week, an ointment composed of :

| | | |
|---|---|---|
| ℞ | Prepared chalk | 10 parts |
| | Coal tar | 1 to 4 parts |
| | Glycerine | 5 parts |
| | Simple cerate | 50 parts |

**For Phthisis.**—

| | | |
|---|---|---|
| ℞ | Chloride calcium | gr. x |
| | Water | ℥ j |
| | Glycerine | ʒ j |

Mix. Give in wine glass full of milk three times a day.

**Eucalyptol as an Antiseptic.**—Eucalyptol may prove to be the antiseptic of the future. Drs. McIntyre and Bauer recently used it in a spray in a modified form of Lister's dressing in an operation for mammary cancer with the very best results.—*St. Louis Clinical Record.*

